# mRNA Vaccine Nanoplatforms and Innate Immunity

**DOI:** 10.3390/v16010120

**Published:** 2024-01-14

**Authors:** Lai Wei, Chunhong Dong, Wandi Zhu, Bao-Zhong Wang

**Affiliations:** Center for Inflammation, Immunity & Infection, Institute for Biomedical Sciences, Georgia State University, Atlanta, GA 30303, USA; lwei11@student.gsu.edu (L.W.); cdong@gsu.edu (C.D.); wzhu3@gsu.edu (W.Z.)

**Keywords:** mRNA vaccine, innate immunity, lipid nanoparticles, polymer nanoparticles, adjuvants

## Abstract

mRNA-based vaccine technology has been significantly developed and enhanced, particularly highlighted by the authorization of mRNA vaccines for addressing the COVID-19 pandemic. Various biomaterials are developed in nano-scales and applied as mRNA vaccine delivery platforms. However, how these mRNA nanoplatforms influence immune responses has not been thoroughly studied. Hence, we have reviewed the current understanding of various mRNA vaccine platforms. We discussed the possible pathways through which these platforms moderate the host’s innate immunity and contribute to the development of adaptive immunity. We shed light on their development in reducing biotoxicity and enhancing antigen delivery efficiency. Beyond the built-in adjuvanticity of mRNA vaccines, we propose that supplementary adjuvants may be required to fine-tune and precisely control innate immunity and subsequent adaptive immune responses.

## 1. Introduction

Message RNA-based vaccines have gained significant prominence since the breakout of the SARS-CoV-2 pandemic. The first FDA-approved mRNA vaccines, developed by Pfizer/BioNTech and Moderna, have revealed a promising avenue for the development of safe and effective mRNA vaccines. These groundbreaking vaccines utilize lipid nanoparticle (LNP) delivery platforms, which deliver nucleotide-modified mRNAs encoding specific antigen proteins (such as the spike proteins of SARS-CoV-2) into the host cell cytoplasm, leading to antigenic protein expression [1,2]. Furthermore, researchers are exploring various biomaterial platforms as carriers for delivering antigenic mRNA, including natural or artificially synthetic large-molecule polymers, modified lipids, and polymer-lipid hybrid structures [3]. These delivery platforms can protect and deliver the mRNAs efficiently, and they have the potential to exert precise control and program over immune responses in vaccine design and therapeutic drug delivery.

**Figure 1:**
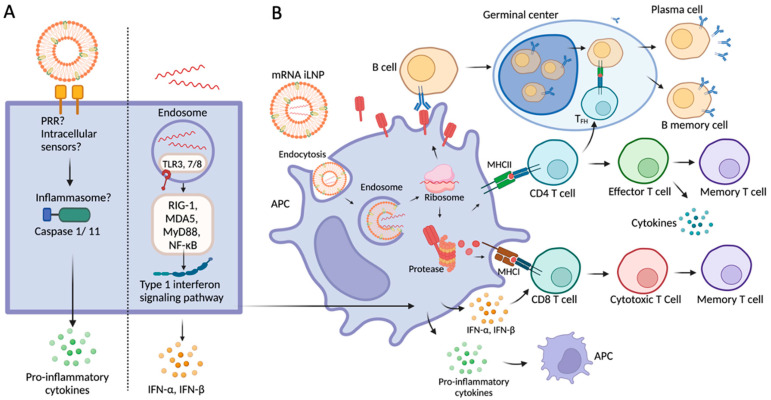
mRNA iLNP vaccine-induced immune response. (**A**) Innate immune response induced by iLNP and mRNA, separately. (**B**) Adaptive immune response induced by mRNA iLNP. PRR, pattern recognition receptor; Nlrp3, NOD-, LRR- and pyrin domain-containing protein 3; ASC, apoptosis-associated speck-like protein containing a caspase recruitment domain; TLR, toll-like receptor; RIG-1, retinoic acid-inducible gene 1; MDA5, melanoma differentiation association protein 5; NF-*κ*B, nuclear factor- *κ*B; MyD88, myeloid differentiation marker 88; T_FH_, T follicular helper cell; IFN, interferon; IL, interleukin. The figure was created with BioRender.com and was last accessed on 13 January 2024.

Message RNA vaccines use the host cellular machinery to produce encoded antigenic proteins to elicit immune responses (Figure 1). Notably, the innate immune system plays an essential role in the initiation and subsequent direction of adaptive immune responses [4]. Both the extraneous mRNA and the iLNP (lipid nanoparticles containing ionizable cationic lipid) delivery systems can induce innate immune responses, thus modulating adaptive immune responses as well as inflammation reactions (Figure 1A). Upon entry into cells, mRNA is released into the cytosol and translated to protein antigens. Antigenic proteins secreted extracellularly or on the cell membrane are recognized by cognate B cells and induce strong germinal center reactions, including maturation and differentiation of B cells, benefiting the generation of antibodies and B memory cells. In addition, antigen proteins possessed into peptides by protease are presented to CD4 or CD8 T cells via MHC molecules. Activated CD4 T cells can differentiate into effector T cells, and T_FH_ helps B cell differentiation in the germinal center. CD8 T cells can also be induced by type 1 interferons and differentiate into cytotoxic and memory T cells (Figure 1B).

However, despite the enormous advances in mRNA vaccine development, our understanding of the relationship between mRNA-based vaccine platforms and the innate immune responses they trigger is not yet complete. This review aims to provide an overview of recent advancements in mRNA vaccine delivery technologies, particularly in the lipid-mRNA and polymer-based mRNA nanoparticles, explore their potential mechanisms in generating innate immune responses, and examine their subsequent impact on inflammation and adaptive immunity.

## 2. Innate Immunity

The innate immune system serves as the first line of host defense against invading pathogens. The non-specific characteristics of the innate immune system prevent most threats as soon as it detects danger signals. Moreover, the innate immune system plays an essential role in bridging and directing the adaptive immune response—a delayed but highly specific and robust immune response that limits the existing infection [4]. An adaptive immune response is triggered by antigen peptides presented on antigen-presenting cells (APCs), such as dendritic cells (DCs) and macrophages. The alteration of innate immune responses can change the course and bias of adaptive immune events, including immune magnitude and potency of the adaptive immunity and immune memory [5,6]. Thus, to achieve an enhanced, preferred, and prolonged immune response and efficient protection, it is critical to study the early association between the innate and adaptive immune systems. This interaction helps limit inflammation while enhancing protective antibody and cellular immune responses.

## 3. mRNAs and Innate Immunity

Message RNA vaccines have gained considerable prominence due to their advantages, including fast and scalable generic manufacturing, flexible sequence modification processes, easy adaptable ability, and superior safety versus DNA vaccines [7].

In mRNA vaccines, mRNA functions as both the guide for immunogen generation and adjuvants. Upon entry into cells, the exotic mRNAs can be sensed by intracellular innate RNA sensors, eliciting an innate immune response [8]. Notably, endosomal toll-like receptors (TLRs), specifically TLR3, TLR7, and TLR8, located within macrophages, dendritic cells, or B cells, are critical viral RNA pathogen recognition receptors (PRRs). They activate NF-*κ*B and MyD88 pathways [9]. Additionally, cytosolic RNA sensors, like the RIG-1-like receptors (RLRs) and MDA5, collaborate with mitochondrial antiviral signaling protein (MAVS), resulting in proinflammatory cytokine production [10]. These innate RNA sensors recognize ligands of single-strand RNA (ssRNA) or double-strand RNA (dsRNA), which trigger robust intracellular signaling pathways, such as the type 1 interferon pathway [11]. Conventional synthetic mRNA can cause potent inflammation and exhibit suboptimal translation efficiency. Activation of numerous innate sensors may result in uncontrolled local and systemic inflammation and down-regulation of antigen expression from mRNA [11,12].

The stability and translation efficiency of in vitro transcribed (IVT) mRNAs have been improved in past decades. The polyuridylic acid (PolyU) sequence in synthesized mRNAs functions as an agonist in TLR recognition, inducing a high level of interferon secretion [13,14]. Pioneering work by Dr. Katalin Karikó et al. found that chemical modification of nucleotides enhanced protein translation levels [15] and shielded IVT mRNA from most innate sensors [16,17]. By replacing uridine with naturally modified pseudouridine Ψ or other derivatives, like N1-methylpseudouridine (m1ψ), 2-thiouridine, 5-methyluridine, and 5-methylcytidine, researchers achieved a remarkable suppression of RNA recognition by endosomal TLR3 and TLR7/8 and cytosolic RIG-1. This strategic modification enabled the precise control of innate immune activation while significantly extending the lifespan of IVT mRNAs [16,17]. Also, the protein translation efficiency is robustly enhanced due to the increased stability of pseudouridine-modified IVT mRNAs and reduced interaction between mRNA and RNA-dependent protein kinase (PKR) [16,17].

The nucleotide-modified mRNA strategy reduces the attack from innate immune systems and increases the probability of protein translation. These breakthroughs in modified nucleic acids provide the critical success of current FDA-approved COVID-19 vaccines [11].

## 4. mRNA Delivery Platforms

Despite the advantages of mRNA vaccines, the intrinsic negative charges and exotic toxicity associated with nucleic acids pose a hindrance to their efficient uptake by host cells, presenting a formidable challenge in the effective in vivo delivery of mRNAs and necessitating the development of suitable delivery platforms. Recent advances in material sciences have yielded promising delivery platforms that facilitate the efficient delivery of mRNA vaccines. The lipid- and polymer-based mRNA vaccine platforms are the most promising and developed ones. A brief comparison of the induced immune responses between these two platforms is shown in Table 1. Herein, we will review the recent advancements in these platforms and the associated innate immunity.

### 4.1. Lipid mRNA Nanoparticles

Lipid nanoparticles mostly contain four kinds of lipids: (1) cationic or ionizable cationic lipids, such as DOTMA, DOTAP, SM-102, or ALC-0315; (2) phospholipids, such as DPPC, DPPE; (3) lipid-anchored polyethylene glycol (PEG), such as DMG-PEG2000 and (4) cholesterol [3]. Cholesterol and phospholipids are structural supports and stabilizers for nanoparticle formulation. These lipids are widely present in cell membranes, are generally non-inflammatory, and do not trigger innate immune recognition. However, the inclusion of other components into lipid nanoparticles, such as cationic or ionizable cationic lipids and PEGylated lipids, have mild cell toxicity and the potential to induce inflammation [3,25].

Dr. Pieter Cullis’s research on ionizable cationic lipids has brought promising prospects for significantly reducing inflammation [25,26]. These ionizable lipids exhibit pH-sensitive properties, being protonated and carrying positive charges at low pH levels. Compared to the permanent cationic liposome surface, ionizable liposomes are neutral in the bloodstream, minimizing off-target interactions with the anionic cell membrane of blood cells [19]. In the endosomal environment, where the pH ranges from 4.5 to 6.5, ionizable lipids undergo protonation, resulting in positive charges on the liposome surface. Consequently, ionizable lipids help to achieve endosomal escape and membrane disruption. The most renowned ionizable lipids are SM-102 and ALC-0315, which have been applied in FDA-approved COVID-19 vaccines.

Lipid nanoparticles containing ionizable cationic lipids are proven to have adjuvant effects in generating high adaptive immune responses after mRNA iLNP vaccination [18]. Dr. Mohamad-Gabriel Alameh’s group studied the adjuvanticity of an iLNP formulation proprietary to Acutas Therapeutics in a mouse model, with recombinant influenza hemagglutinin (rHA) as the antigen [3,27,28]. Their results showed that the rHA protein adjuvanted with empty iLNPs induced robust IL-6 secretion within 24 h after intramuscular injection, indicating the activation of follicular T cells in draining lymph nodes. In addition, HA mRNA iLNP vaccines exhibited the capacity to induce similar levels of germinal center reactions to those achieved with iLNPs carrying rHA protein, resulting in the activation of follicular T cells (CXCR5+, Bcl6+) and antigen-specific B cells (Fas+, Gl7+). Ionizable cationic lipid-containing LNPs played a significant role in enhancing antigen-specific antibody responses, including neutralizing antibody responses, during vaccination, whereas LNPs without ionizable cation lipids failed to induce a neutralizing antibody response [27].

Dr. Chunfeng Li et al. studied the potential innate sensing mechanisms of SM-102-containing BNT162b2 in aspects of antibody responses and T cell immunity in mice [29]. Effective protection against the antigenic virus hinges on two critical factors: the induction of high levels of neutralizing antibodies for rapid virus infection inhibition and the activation of strong CD8+ cytotoxic T cell response for efficient virus clearance [30]. When administrating BNT162b2 in knockout mice, they found that the elevated antibody response and T cell immunity did not require the Nlrp3 (NOD-, LRR- and pyrin domain-containing protein 3) or ASC (a caspase recruitment domain) dependent inflammasome activation [29]. The TLR2, TLR5, TLR7, or STING- cGas pathways were not also essential for the induction of antibody or T cell response. However, the MDA5-IFNAR1 signaling pathway was found to play an important role by inducing type 1 IFN response, which is responsible for strong CD8+ T cell responses [29].

Nevertheless, mRNA iLNPs activate the innate immune system and may cause inflammation and side effects in vaccinated individuals [31]. Despite the mitigated toxicity with ionizable lipids, acute inflammatory responses, including allergy, muscle pain, and fever, have emerged in adults and children after the intramuscular administration of COVID-19 mRNA vaccines [32,33]. Rare disease cases, such as myocarditis, were reported after COVID-19 vaccination [34]. Approximately 1 to 5 myocarditis cases were reported among 1 million COVID-19 mRNA vaccinations within the general population [35,36]. Ionizable cationic lipid-containing LNPs are recognized by innate immune cells, producing a variety of pro-inflammatory effectors, including chemokines and cytokines, which contribute to local and systemic inflammation [27,29,37]. Cytokine levels at local tissue, where iLNPs were injected, such as interleukin-1β (IL-1β) and interleukine-6 (IL-6), were significantly increased. Chemokines, such as CCL2, CCL3, CCL4, CCL7, and CCL12, were greatly induced and helped recruit and activate neutrophils and monocytes [37].

Ionizable cationic lipid-containing LNPs induce pro-inflammatory cytokines via several possible mechanisms [18]. One such mechanism could involve inflammasome activation. Dr. Ndeupen et al. found that empty iLNPs proprietary to Acuitas Therapeutics induced a robust production of IL-1β in murine bone-marrow derived macrophages. However, the IL-1β production was significantly inhibited in caspase 1/11 double deficient murine macrophages [38]. In another study, Dr. Ndeupen et al. injected the mRNA-iLNPs intradermally in mice and detected upregulation of transcripts associated with the activation of inflammasomes such as IL-1β and Nlrp3 (NOD-, LRR- and pyrin domain-containing protein 3) [37]. Other proposed hypotheses include mechanisms that can sense the membrane fusion of iLNPs and plasma or endosomal membrane, which also lack direct evidence [39]. In addition, iLNPs may be sensed directly by innate sensors or indirectly via innate immune activation. A variety of PRRs or intracellular sensors may be involved in the recognition of iLNPs, but this remains unclear [18].

Owing to limited studies, currently available data cannot capture the full picture of the iLNP-induced innate immunity, leaving room for further investigation and a more comprehensive exploration of the underlying factors and pathways involved. How iLNPs are sensed by the innate immune system and how they trigger innate immune responses are still major questions remaining in mRNA vaccine research.

### 4.2. Polymer-Based mRNA Nanoparticle

As alternative nanocarriers for mRNA vaccines, polymers are easier to synthesize than lipids, more flexible to modify, and more stable. They have been widely applied for DNAs and RNAs in vitro transfection studies and in vivo drug deliveries. Polymers can be recognized universally by various TLRs (particularly TLR2, TLR4, TLR6) on dendritic cells. Multiple activated TLR/MyD88 signaling pathways in dendritic cells lead to Th1 response and elicit CD8+ T cell response in the host [40,41].

Poly(ethylenemine) (PEI) is one of the most widely studied cationic condensed polymers. One appreciated feature of PEI is that it enables the payload to achieve endosomal escape. PEI polyplexes enter the cell via endocytosis and accumulate in cell compartments, such as endosomes. PEI contains numerous condensed positively-charged nitrogen atoms, acting like a proton sponge [42]. During acidification of endosomes, the high buffering capacity of PEI can cause elevation of osmotic pressure and endosomes to burst, which eases the safe release of internal nucleic acids to cell cytoplasm [43]. PEIs have been widely studied by various molecular lengths (from 2 k to 50 k), in vitro transfection efficiency, and toxicity. The research on PEI-siRNA nanoparticles reveals that PEI is a potent TLR5 agonist. PEI encapsulating nontargeting siRNAs can stimulate TLR3 and TLR7, are preferred to be engulfed by DCs, and can program PD-1 cell death in ovarian cancer-associated DCs, which may facilitate antitumor immunity if therapeutic siRNAs are encapsulated [21].

PEIs are usually modified to enhance transfection efficiency and mitigate intrinsic toxicity. Poly D, L-lactide-co-glycolic acid (PLGA) is commonly used for PEI modification. PLGA is a nontoxic polymer and can be metabolized during the tricarboxylic acid cycle [44]. Due to its biodegradable and biocompatible ability, PLGA is often utilized for polymer or polymer nanoparticle outer layer modification. For instance, PLGA-modified PEI encapsulating mRNAs encoding green fluorescent protein (GFP) targeted monocyte-derived DCs and induced efficient and prolonged in vitro transcribed protein stimulation [45]. A self-assembled vitamin E succinate-modified PEI micelle for mRNA delivery enhanced mRNA transfection efficiency in different cell lines in vitro and induced high serum IgG antibody titers and IFN-γ and IL-4 secreting T cell response [22].

Poly (beta-amino esters) (PbAEs) are promising alternative polymers for PEIs in gene delivery due to their high biodegradability and considerable flexibility of chosen monomers. PbAEs are synthesized by a straightforward Michael addition reaction of amines and diacrylates and end-capped by a diamine [46,47,48]. The facile synthesis of PbAEs gives a widespread polymer library with flexible modifications. With a cationic feature, PbAEs are suitable for electrostatic condensation with negatively charged nucleic acids. They were reported to have much lower cytotoxicity than common gene delivery reagents, such as lipofectamine and PEIs [49,50].

The interaction of PbAE polymers and innate immune cells has been studied. PbAE nanoparticles can activate DCs strongly and enhance antigen-presenting ability, significantly increasing immune activation in mouse lymph nodes, whereas free/soluble PbAE is limited in dendritic cell activation [51]. Also, molecular weights and degradation levels of PbAEs were found to impact the activation level by analyzing DC markers (CD40, CD80, CD86, MHCII) [52]. In the RAW264.7 cell model, macrophages can be activated by both free and particulate forms without inducing TLR activities or activating the NF-*κ*B signaling pathway. In contrast, interferon-regulatory factor (IRF) pathway activity is involved [53].

PbAEs were found to be efficient for mRNA delivery in gene therapy research. PbAEs generally have a half-life time of 1 to 7 h in the aqueous phase, which enables shielding mRNA delivery during endocytosis and endosome escape and releasing mRNA when it is transported in the cytoplasm [24,54,55]. In relevant research, a new therapeutic platform was designed using mRNA-PbAE nanoparticles in cellular reprogramming and cancer therapy. A T-cell-targeting therapy was created by encapsulating mRNA encoding gene-editing agents (Foxo1) with PbAE 477 and modified polyplexes surface with anti-cancer T-cell antibodies (CD3, CD8) via a polyglutamic acid (PGA) linker [56]. The researchers also used this technique for anti-tumor therapy by reprogramming tumor-associated macrophages into tumoricidal macrophages. They developed a mRNA-PbAE nanoparticle encoding M1-polarizing transcription factors (IRF5, IKKβ), with PGA-conjugated Di-mannose on the surface to shield the positive charges of nanoparticles [57]. These mRNA-PbAE nanoparticle systems precisely target desired cells and transfect cargo mRNAs at a practical expression level.

PbAE-mRNA nanoparticles are proven to achieve protein expression in vivo by using mRNA payload encoding firefly luciferase or fluorescence as signal reporters. When PbAE was co-formulated with PEG-lipid, the nanoparticle stability was improved, and the in vitro expression level of luciferase mRNAs was significantly increased compared to nanoparticles without PEG-lipid. By using luciferase-coding mRNA as a reporter gene for systemic administration, this hybrid polymer-lipid nanoparticle can be delivered efficiently to the lungs [58]. Moreover, noninvasive aerosol inhalation of luciferase mRNA delivery was achieved using a hyperbranched PbAE (hPbAE)-formulated nanoparticle. Twenty-four hours after inhalation of the hPbAE nanoparticles, a high level of luminescence was detected in all five lung lobes but no other tissues [59]. These studies showed that the stability and efficiency of PbAE-related nanoparticles may provide clinical opportunities in lung delivery systems.

## 5. Guided Immunity: Type I IFN Signaling and mRNA Vaccines

Generally, type 1 IFN response is critical to upregulate the anti-viral state and induce effective CD8+ T cell immunity and memory [60]. The immunostimulatory ability of mRNA vaccines may drive strong type 1 IFN signaling and the maturation of dendritic cells, benefiting effector T and B cell activation [61,62]. In contrast, in self-amplifying mRNA research, it was shown that blocking IFN-α/β signaling (early type 1 IFN response) may maximize the amplification of RNA replicon and protein expression of mRNA cargo, with a strong CD8+ T cell response elicited [63]. One explanation is that early type 1 IFN signaling can either stimulate or inhibit the antigen-activating T cell immunity in a time-dependent manner [64,65]. If the T cell receptor activation of CD8+ T cells precedes IFN-α receptor signaling, the proliferation and differentiation of CD8+ T cells can be achieved, with sustained expression of critical genes for T cell memory [66,67]. In contrast, T cell immunity was suppressed when IFN-α/β was exposed to CD8+ T cells before their TCR signaling activation [68].

Despite the advances in mRNA vaccine development, the innate immune pathways activated by mRNA delivery nanoparticles are to be studied further. The activation is uncontrolled and untimed. There is room to build precise and strong control of the induced innate immune responses.

## 6. Adjuvants and Future Direction

Adjuvants play a crucial role in vaccine design to achieve targeted immunity and broader protection. In the context of mRNA vaccine platforms, the adaptable nature of mRNA encoding frames allows for the design of protein adjuvants in mRNA forms, the same as antigens. However, a delicate balance should be considered between adjuvanticity and mRNA expression levels. Different mRNA vaccine formulations and vaccination routes may affect the outcomes of type 1 IFN, emphasizing the need for adjuvants that precisely control type 1 IFN signaling.

An example of an mRNA-encoded adjuvant is Fβ2, a fusion protein combining IFN-β and the ectodomain of TGF-β receptor II. When delivered intratumorally, Fβ2 showed an effective antitumor T cell response by improving the antigen-presenting function of dendritic cells and modulating the ability of myeloid cells to improve CD8 T cell responses [69]. Another study utilized an mRNA-encoded adjuvant with mRNA human papillomavirus (HPV) vaccine to achieve selective activation of type 1 IFN and preserve antigen expression in vivo. The adjuvant involved constitutively active mutations of TLR, IRF3/7, MAVs, and STING signal proteins, with STINGV155M showing strong adjuvanticity by upregulating IFN-γ secretion and inducing higher antigen-specific CD8+ T cell responses [70].

To achieve more precise control over innate immune responses, general adjuvant strategies directing innate immunity pathways may be applied to novel mRNA nanoparticle technologies. TLR agonist adjuvants, for instance, activate antigen-presenting cells quickly through TLR signaling pathways, generating a robust humoral immune response [71]. In addition, cytokines, naturally secreted by the host’s immune system, serve as safer and more reliable adjuvants than synthetic or exogenous adjuvants [72]. The predictable mechanisms of cytokine adjuvants are dependent on their immunomodulation properties. For example, in our lab, we have constructed GIFT4, a fusion protein of GM-CSF and interleukin-4 (IL-4), which improves B lymphocyte proliferation in vitro. When formulated as a GPI-anchored adjuvant in HIV VLPs, the GPI-GIFT4-adjuvanted group exhibited highly elevated IgG and IgA levels at several mucosal sites [73].

For mRNA vaccines, the designs of mRNA-formed adjuvants are accessible and flexible. The protein-formed adjuvants can be quickly constructed and manufactured as protein-encoding mRNAs. The mRNA-formed adjuvants can then be encapsulated into the nanoparticles along with mRNA antigens. This novel adjuvant approach holds promise for integrating the advantages of mRNA delivery nanoparticles and adjuvanticity, offering a fast design and manufacturing process, high immunogenicity of mRNA nanoparticles, and precise and elevated innate immunity from adjuvants.

## 7. Discussion

mRNA vaccine technologies have been a significant advancement in the vaccine development field. Their efficacy and research value have been demonstrated during the COVID-19 outbreak. The FDA-approved COVID-19 mRNA vaccines were believed to have averted tens of thousands of hospitalizations and deaths in the US [74,75]. However, the deep knowledge of the innate immunity of current licensed LNP-mRNA vaccines is still under investigation and will provide more convincing opinions of modifications on either mRNAs or delivery platforms.

iLNPs show high adjuvant effects, especially in speeding up germinal center reaction and enhancing antigen-specific antibody responses. And this strong communication between innate and adaptive immune systems also benefits cytotoxic T cell response and reacting quickly against virus infection. Although the mRNA iLNP vaccine formulations have evolved with lower toxicity than permanently cationic LNPs, they still induce inflammation and side effects when administrated on humans. iLNPs induce increased pro-inflammatory cytokine production at the local administration site and recruit innate immune cells at the site and cause redness and swelling [32,33]. Researchers are striving to develop novel ionizable lipid components to enhance the potency of ionizable lipid nanoparticles (iLNPs) while minimizing off-target side effects.

Owing to the excellent flexibility in molecular structure design and modifications, polymer-based delivery platforms are also promising mRNA delivery platforms and hold massive potential for future mRNA applications. The activation of innate immunity by polymers reveals potential adjuvant effects, in addition to the ability for mRNA shielding and delivery. However, up to date, no commercial polymer-based mRNA vaccines have been developed. The ongoing quest for more potent polymers persists. Moreover, the limited data hinders a comprehensive understanding of the innate immune responses and side effects associated with mRNA polymer vaccines. In-depth studies are needed to address gaps in our knowledge and facilitate the development of polymer platforms.

Novel nanoplatforms that efficiently deliver mRNAs and are of great biocompatible ability and biodegradability are urgently wanted. Also, safe delivery platforms that allow intranasal or intratracheal delivery should be in consideration. As for respiratory viruses that annually cause epidemics, such as influenza viruses, respiratory syncytial viruses (RSVs), and SARS-CoV-2, mucosal mRNA delivery platforms are more favorable, in the aspect of generating mucosal immunity for fast viral clearance.

In conclusion, tuning the innate immune response can modulate the potency and quality of the adaptive immune responses. Studying the early interplay of innate and adaptive immune systems is critical to achieving enhanced, preferred, and prolonged immune response and efficient protection and limiting inflammation.

## Figures and Tables

**Table 1:** Comparison of mRNA delivery platforms.

Type of Nanoparticles	Nanoparticle Formulation	Innate Sensor	Innate Immune Response	Adaptive Immune Response
iLNP	Ionizable cationic lipids, phospholipids, PEG-lipids, and cholesterol	Possible PRRs or intracellular sensors [18]	Induce robust IL-6 secretion within 24 h after intramuscular injection [19].	Activate follicular T cells and antigen-specific B cells in the germinal center [19]. Type 1 IFN signaling responsible for CD8+ T cell responses [20].
Polymer-based mRNA nanoparticles	PEI	TLR3, TLR5, TLR7	Engulfed by dendritic cells and induce MyD88 signaling pathway [21].	Elicit high humoral and cellular response when applied in SARS-CoV-2 vaccine [22].
	PbAE	no TLR activation or NF-*κ*B pathway activation	enabled to be delivered in DCs [23] can activate macrophages via the IRF pathway [24]	With CpG as an adjuvant, can elicit a high level of OVA-specific CD8+ T cell response and long-term CD8+ T cell response in tumor vaccination in mouse melanoma models [23].

## Data Availability

Not applicable.
